# Assessment of knowledge, attitude, and practice regarding statutory rape and its management among healthcare workers in emergency wards in Ibadan, Nigeria

**DOI:** 10.11604/pamj.2025.51.21.47295

**Published:** 2025-05-26

**Authors:** Million Teklay Solomon, Christopher Odianosen Aimakhu, Olugbenga Oluseun Saanu, Ines Nshimirimana, Binta Jallow, Iacane Bampoque, Agness shimilimo, Amos M´yisa Makelele, Edmond Onidje

**Affiliations:** 1 Pan African University Life and Earth Sciences Institute (including Health and Agriculture), Ibadan, Nigeria,; 2 University of Ibadan, University College Hospital, Ibadan, Nigeria

**Keywords:** Statutory rape, healthcare workers, knowledge-attitude-practice (KAP), emergency care, Nigeria

## Abstract

**Introduction:**

statutory rape is a critical public health and legal issue with severe consequences for survivors in Nigeria and is related to insufficient healthcare training, hindering effective case management. This study assessed the knowledge, attitudes, and practices (KAP) of healthcare workers in emergency wards in Ibadan to identify gaps and challenges in statutory rape management.

**Methods:**

a cross-sectional study was conducted among 198 healthcare workers in Ibadan, using structured questionnaires and analyzed with descriptive statistics, t-tests, and chi-square tests.

**Results:**

the study revealed significant gaps in healthcare workers' KAP concerning statutory rape. While 81.3% of healthcare workers were aware of the term “statutory rape,” only 75.3% understood the legal provisions related to it, such as the legal age of consent. Doctors had significantly higher KAP scores than nurses in all domains: knowledge (p= 0.0029), attitude (p= 0.0044), and practice (p= 0.0028). Despite recognizing the severity of statutory rape (76.3%), 33.3% of healthcare workers had never managed such cases, and many reported infrequent encounters with statutory rape cases. A significant proportion (65.2%) of healthcare workers identified inadequate forensic training as a major barrier, while (83.3%) cited legal and reporting constraints, and (31%) mentioned geographical access limitations. The most commonly reported physical and psychological symptoms observed in survivors included genital injuries (93.4%), psychological trauma (86%), STDs and infections (81.3%), bleeding (85.4%), and extra-genital injuries (69.2%).

**Conclusion:**

this study highlights deficiencies in healthcare workers´ knowledge and preparedness in statutory rape case management. Strengthening forensic training, legal awareness, and healthcare-legal collaboration is essential to improving response effectiveness.

## Introduction

Sexual violence, particularly rape, is a grave violation of human rights with serious physical, psychological, and social consequences [1]. Despite legal frameworks, it remains widely underreported due to stigma, fear of retaliation, and ineffective law enforcement [2]. Adolescent girls are especially vulnerable, often experiencing sexual violence as their first encounter with sexual activity [3]. This, with the weak legal systems and the societal taboos, more often than not protects the perpetrators while leaving the survivors with negligible medical, psychological, or legal support [4,5]. Statutory rape refers to sexual activities that involve a minor below the age of consent and is an important public health and legal issue [6]. While legal definitions vary across jurisdictions, it is widely recognized as a form of sexual violence with serious consequences, including unintended pregnancies, sexually transmitted infections (STIs), and profound psychological distress [7,8]. The issue is exacerbated by inadequate legal enforcement, healthcare system gaps, and persistent societal silence [9]. Globally, sexual violence affects 12% to 25% of adolescent girls, with perpetrators often being family members, acquaintances, or trusted figures [4]. In Nigeria, the situation is particularly alarming. Among out-of-school female adolescents, 15.8% reported forced sexual initiation, while 18% of sexually active respondents experienced rape [10]. Indeed, one such study found that out of the sexual violence experienced by 39.5% of women between 17-24 years old, most perpetrators were familiar individuals [11]. It is even worse for vulnerable populations, such as female child laborers, with risks as high as 77.7% who reported experiences of sexual violence in Maiduguri [12], while in the same region, 51.3% of female university students reported similar incidents [13].

One rural healthcare facility in Northwest Nigeria recorded 3% sexual assault among gynecological consultations, where 91.7% of the cases were minors below the age of 16 years [14]. Healthcare professionals are crucial in managing statutory rape cases, offering emergency medical care, forensic evidence collection, and psychosocial support [15]. Essential interventions include STI prophylaxis, emergency contraception, and psychological counselling [2]. However, in low- and middle-income countries, several barriers hinder effective intervention. Most healthcare providers are not sufficiently trained to identify, document, and report statutory rape cases; hence, there is always delayed support for survivors, with further weakened legal actions [9,16]. Very few studies in Nigeria, particularly in Ibadan, have assessed emergency healthcare preparedness to respond to such incidents [17]. These, together with inconsistent medical practices, insufficient legal knowledge, and stigma associated with the incidents, further contribute to a fragmented response system. High-profile cases from medical professionals, educators, and public figures raise awareness of a pressing need in terms of systematically addressing statutory rape through healthcare [17]. The failure to have prescribed protocols often sets the stage for incomplete medical record-keeping, delayed care, and weakened justice [18]. Enhancing acute healthcare responses for such cases, therefore, requires clear policies, better training of medical officers, and a legal-medical interface. This study has aimed at assessing emergency healthcare responses to statutory rape in Ibadan through evaluating knowledge, awareness, and preparedness of healthcare professionals; identifying challenges related to case identification, medical intervention, and cases reporting.

## Methods

**Study area:** this study was conducted in Ibadan, the capital and largest city of Oyo State, Nigeria. Ibadan is one of the most populous cities in Nigeria, with an estimated 4.04 million people, and is also a major healthcare hub in southwestern Nigeria [19]. The research was conducted across three hospitals: University College Hospital (UCH) Ibadan, Adeoyo Maternity Teaching Hospital Ibadan, and Oluyoro Catholic Hospital Ibadan. These hospitals had been selected because they have well-recognized emergency departments where statutory rape cases are likely to be managed, hence representative samples of health workers who work in the emergency care section.

**Study design:** a cross-sectional research design was employed in this study to assess the knowledge, attitudes, and practices (KAP) of healthcare workers regarding statutory rape and its management in emergency departments. A mixed-methods approach was utilized, combining both quantitative and qualitative data collection techniques. This approach enabled a comprehensive exploration of healthcare workers' responses to statutory rape cases, ensuring that both numerical data and in-depth personal insights were captured.

**Study population:** the target population included healthcare workers from the emergency departments of University College Hospital Ibadan, Adeoyo Maternity Teaching Hospital Ibadan, and Oluyoro Catholic Hospital Ibadan. The study focused on two primary categories of healthcare providers who deliver critical services: emergency medical doctors (EMDs) and nurses (SNs). These healthcare professionals were chosen due to their direct involvement in the management of sexual assault cases, particularly statutory rape, making them the appropriate respondents for assessing the KAP related to such cases. The study captured a broad spectrum of healthcare workers´ perspectives on the management of statutory rape, focusing on both doctors and nurses.

**Sampling and sample size calculation:** a purposive sampling technique was employed to select the three hospitals involved in the study. Within each hospital, healthcare workers from the emergency departments were selected using a simple random sampling method to ensure a representative sample. To calculate the appropriate sample size, the Leslie Kish formula for estimating proportions in cross-sectional studies was used.


$$n_{0}=\frac{zx^{2}PQ}{d^{2}}$$

Where n_0_ is the required sample size, Z is the Z-value at a 95% confidence level (1.96), P is the estimated prevalence of adequate knowledge on statutory rape among healthcare workers (0.417) based on Abeid *et al*. [20], Q=1- P, and d the margin of error (0.05). The sample size calculation resulted in 374 respondents. To account for potential attrition or non-responses, a 5% increase was added to the sample size, bringing the calculated sample size to 393 respondents. However, since the total population of healthcare workers in the selected hospitals was approximately 400, the sample size was further adjusted using the finite population correction formula. The finite population correction formula is expressed as:


$$n_{0}=\frac{n_{c}}{1+\frac{n_{c}}{N}}$$

Where n_c_=393 (the calculated sample size) and N=400 (the total population of healthcare workers in the selected hospitals). After applying the formula, the final adjusted sample size was determined to be 198 respondents. This adjustment was made to ensure a representative sample without over-sampling, thus maintaining statistical accuracy while considering the small population size.

**Data collection procedure:** data collection took place between July and September 2024 and was carried out using structured questionnaires. The questionnaire was designed to assess the knowledge, attitudes, and practices (KAP) of healthcare workers regarding statutory rape, including demographic information, work experience, and specific knowledge related to statutory rape management. The questionnaire was pre-tested with a small group of healthcare workers from a similar setting to ensure its clarity, relevance, and reliability before it was administered to the full sample.

**Measurement of knowledge, attitude, and practice (KAP):** knowledge was assessed by assigning one point for each correct response and zero for incorrect or uncertain answers, with scores expressed as a proportion of the maximum possible. Attitude was measured using a 5-point Likert scale, with higher scores indicating more favorable attitudes toward statutory rape case management. Practice was evaluated based on adherence to best practices in case handling, with higher scores given to appropriate responses.

**Data analysis:** the data were entered into Microsoft Excel® 2019 and analyzed using STATA software. Descriptive statistics were used to summarize the data, with continuous variables reported as means and standard deviations (mean ± SD) and categorical variables as frequencies and percentages. Independent t-tests were performed to compare mean KAP scores between doctors and nurses. All analyses were performed at a 95% confidence level.

**Ethical considerations:** ethical approval was obtained from the University of Ibadan/University College Hospital Ethical Committee (UI/UCH EC) and the Oyo State Ministry of Health (Ref: AD 13/479/421). Furthermore, written informed consent was obtained from all participants, ensuring confidentiality and guaranteeing their right to withdraw at any time without penalty. Moreover, all data were anonymized, securely stored, and restricted to the research team to maintain privacy and security.

## Results

**Sociodemographic characteristics of the participants:** the study examined the demographic and work-related characteristics of doctors and nurses, highlighting key differences between the two professions (Table 1). Most respondents had less than five years of experience (56%), with a higher proportion among nurses (37.7%) than doctors (17.6%). The gender distribution showed a predominantly female workforce (74.2%), with nurses (60%) having a higher representation than doctors (15%). Marital status data indicated that 33.3% of respondents were married, with a higher percentage among nurses (39.2%) than doctors (22.1%). Christianity was the dominant religion (79.8%), followed by Islam (19.7%)., Yoruba was the most represented ethnic group (84%), particularly among nurses (61%) compared to doctors (24%). In terms of education, the majority of respondents held a bachelor´s degree (66.7%), more common among doctors (28%) than nurses (39%), while a smaller proportion had a master´s degree (8%) and only one respondent (0.5%) had a PhD. Earnings varied significantly, with 31% earning between ₦100,000 - ₦200,000, mainly nurses, while 19.2% earned below ₦100,000, and doctors (19.1%) were more likely to earn above ₦300,000 compared to nurses (3%). Hospital distribution showed that most respondents worked at University College Hospital (UCH) (49%), where doctors (27%) were concentrated, while nurses were more evenly distributed across Catholic (23%) and Adeoyo Maternity (26%) hospitals.

**Table 1:** socio-demographic characteristics of healthcare workers in Ibadan (2024)

Variables	Factor	Doctor (n = 68)	Nurse (n = 130)	Total (n = 198)
Work experience (years)	≥25 years	0 (0.0%)	2 (0.8%)	2 (0.5%)
	11-15 years	11 (5.9%)	25 (13.1%)	36 (18.2%)
	16-25 years	3 (1.5%)	11(6.2%)	14 (7.1%)
	5-10 years	19 (10.3%)	17 (9.2%)	36 (18.2%)
	Less than 5 years	35 (17.6%)	75 (37.7%)	110 (56 %)
Marital status	Married	43 (22.1%)	77 (39.2%)	120 (33.3%)
	Single	25(13.2%)	53 (26.9%)	78 (22.2%)
Gender distribution	Male	39 (19.7%)	12 (6.06%)	51 (25.7%)
	Female	29 (14.6%)	118 (59.6%)	147 (74.2%)
Religious affiliation	Christian	54 (79.4%)	104 (80.0%)	158 (79.8%)
	Muslim	13 (19.1%)	26 (20.0%)	39 (19.7%)
	Other	1 (1.5%)	0 (0.0%)	1 (0.5%)
Ethnicity	Yoruba	51 (24%)	115 (61%)	166 (83.8%)
	Igbo	14 (20.6%)	10 (7.7%)	24 (12.1%)
	Other	3 (4.4%)	5 (3.8%)	8 (4.0%)
Education level	Bachelor’s degree	55 (28%)	77 (39%)	132 (66.7%)
	Master’s degree	3 (2 %)	13 (7%)	16 (8.08%)
	Other qualifications	10 (5.5%)	39 (20%)	49 (25%)
	PhD	0 (0.0%)	1 (0.51%)	1 (0.51%)
Monthly earnings (₦)	Less than 100,000₦	3 (1.5%)	38 (19.2%)	41 (20.7%)
	100,000₦ - 200,000₦	8 (4.04%)	62 (31.3%)	70 (35.5%)
	200,000₦ - 300,000₦	20 (10.1%)	24 (12.1%)	44 (22.2%)
	Above 300,000₦	37 (18.6%)	6 (3.03%)	43 (22%)
Hospital distribution	Adeoyo maternity hospital	11 (6%)	40 (20.2%)	51 (26%)
	Catholic hospital	4 (2.02%)	46 (23.2%)	50 (25.2%)
	University college hospital (UCH)	53 (27%)	44 (22.2%)	97 (49%)

**Knowledge and perception of statutory rape among healthcare workers in Ibadan (2024):** the majority of healthcare workers (81.3%) reported having heard of the term statutory rape, with a higher proportion of nurses (52%) than doctors (28.3%) indicating awareness (Table 2). However, (19.2%) of respondents were unfamiliar with the term. The most commonly cited sources of information among healthcare workers were social media (60.2%) and mass media (35.4%), followed by academic articles (22.0%), magazines (7.0%), friends (9.3%), and formal education (19.3%). A significant proportion of healthcare workers (75.3%) correctly identified 18 years as the legal age of consent for sexual intercourse in Nigeria, while 25.2% either incorrectly stated a lower age or admitted to being unsure. When asked whether sexual intercourse with minors is perceived as normal in society, (16.2%) of respondents believed that it was accepted, while (84.3%) disagreed. Only (36.1%) of healthcare workers were aware of the 'Romeo and Juliet' law, which provides legal exceptions for consensual sexual activity between minors. Awareness was slightly higher among nurses (23.0%) compared to doctors (13.1%), while 64.1% of all respondents had never heard of the law. Less than one-third of respondents (29.3%) confirmed knowledge of the existence of laws against statutory rape in Nigeria, while 45.0% were unsure. A further 26.4% incorrectly stated that no such laws exist. Among those who were aware of statutory rape laws, 60.0% reported learning about them through mass media, 23.0% through law enforcement (police), and 23.0% through word of mouth. A total of 26.1% of healthcare workers believed that certain cultural or religious practices in Ibadan support child marriage, while 74.2% disagreed.

**Table 2:** knowledge and perception of statutory rape among healthcare workers in Ibadan

Variables	Response	Doctors (n = 68) (n, %)	Nurses (n = 130) (n, %)
Awareness of the term 'Statutory Rape	Yes	56 (28.3%)	104(52%)
	No	12 (6.1%)	26(13.1%)
Primary sources of information on statutory rape	Mass media	23 (33.82%)	34(26.15%)
	Social media	23 (33.82%)	74(56.92%)
	Academic articles	18 (26.47%)	17 (13.07%)
	Magazines	8 (11.76%)	3 (2.39%)
	Friends	8 (11.76%)	7 (5.38%)
	Formal education (college/university)	18(26.47%)	13(10%)
Knowledge of the legal age of consent in Nigeria	Correct response (≥18 years)	57 (29.0%)	92 (46.5%)
	Incorrect response (<18 years)	11 (6.0%)	38 (19.2%)
Perceived societal acceptance of sexual intercourse with minors	Yes	10 (5.1%)	22 (11.1%)
	No	58 (29.3%)	108 (55.0%)
Awareness of the 'Romeo and Juliet' law	Yes	26 (13.1%)	45 (23.0%)
	No	42 (21.2%)	85 (43.0%)
Awareness of statutory rape legislation in Nigeria	Aware of statutory rape laws	13 (7.0%)	44 (22.3%)
	Unaware of statutory rape laws	22 (11.2%)	30 (15.2%)
	Unsure	33 (17.0%)	55 (28.0%)
Primary sources of legal knowledge on statutory rape	Media	25 (58 %)	59(45.38%)
	Law enforcement (police)	4 (21.1%)	15(11.53%)
	Word of mouth	7 (9 %)	11(8.46%)
Perceived cultural or religious support for child marriage	Yes	23 (12.0%)	28 (14.1%)
	No	45 (23.0%)	102 (52.0%)

**Healthcare workers´ experience, attitudes, and challenges in managing statutory rape cases in Ibadan (2024):** a total of 151 (76.3%) of healthcare workers considered statutory rape a serious issue, while 80.3% emphasized the importance of confidentiality and privacy in handling such cases (Table 3). Among respondents, 33.3% had managed at least one case of statutory rape in the emergency ward, with more nurses (54) than doctors (32) reporting such experience. The frequency of statutory rape cases varied, with 25.3% of respondents that have managed it stating they rarely encountered such cases, 16% reporting quarterly cases, (4%) seeing cases monthly, 10.3% annually, 3% weekly, and 1% fortnightly. The most commonly reported physical and psychological symptoms observed in survivors included genital injuries (93.4%), psychological trauma (86%), STDs and infections (81.3%), bleeding (85.4%), and extra-genital injuries (69.2%). Other clinical indicators included smears of buccal, vaginal, and rectal mucosa (46%), clothing evidence (37%), combed samples of scalp and pubic hair (32%), fingernail clippings and scrapings (35%), and the presence of semen (31%). Regarding comprehensive care practices, 76.3 % of healthcare workers stated that treating the survivor was a key part of care, while 69.2% emphasized the importance of examining the survivor, 62.1% highlighted the need to ensure survivor safety, 71.2% reported testing the survivor, and 55.1% recorded evidence of injuries. The most commonly reported challenges in providing care included reporting and legal issues (83.3%), lack of specialized training for service providers (65.2%), and geographical barriers such as transportation (31%). When asked about triage priority for statutory rape survivors in emergency care, 69.2% of respondents disagreed with assigning them a green triage category and waiting like other patients, while 31% agreed.

**Table 3:** healthcare workers´ attitudes, practices, and challenges in statutory rape care in Ibadan

Variables	Response	Doctors (n = 68) (n, %)	Nurses (n = 130) (n, %)	Total (n = 198)
Attitudes toward statutory rape cases	Believe statutory rape is a serious issue	57 (84.3%)	94(72.30%)	151 (76.26%)
	Believe confidentiality and privacy should be prioritized	63 (92.64%)	96 (73.84)	159 (80.30%)
	Recognize the impact of family and societal barriers	55 (80.3%)	118 (90.76	173(87.37%)
	Acknowledge psychological trauma and stigma	56 (82.3%)	76 (58.46%)	132(66.66%)
Clinical practices in managing statutory rape survivors	Have managed a statutory rape case	32 (16.4%)	54 (28%)	86 (33.3%)
	Have not managed a statutory rape case	33 (17%)	76 (39.0%)	109(67%)
Frequency of encountering statutory rape cases	Rarely	7(10. 29%)	43 (33.84%)	50 (25.25%)
	Weekly	0 (0.0%)	5 (6.66%)	5(2.52%)
	Monthly	1 (4.0%)	7 (6.0%)	8 (4.04%)
	Quarterly	1 (6.0%)	12 (10.0%)	13(16.0%)
	Yearly	2 (3.1%)	7 (7.3%)	9(10.34)
	Fortnightly	1 (1.0%)	1 (2.0%)	2(1.01%)
Clinical symptoms observed in survivors	Genital injury	59 (86.76%)	126(96.92%)	185(93.43%)
	Extra-genital injury	48 (70.58%)	89 (67.69%)	137 (69.19%)
	Psychological trauma	58 (85.29%)	112 (86.15%)	170 (85.85%)
	STDs and infections	54 (79.410%)	107(81.53%)	161(81.31%)
	Bleeding	57 (83.82%)	112 (86.15%)	169 (85.35%)
	Clothing evidence	30 (44.11%)	43 (33.07%)	73 (36.86%)
	Smears of buccal, vaginal, and rectal mucosa	35 (51.47%)	56 (43.97%)	91(45.95%)
	Combed hair samples (scalp/pubic)	25 (36.76%)	38 (23.75%)	63(31.81%)
	Fingernail clippings/scrapings	28 (41.17%)	41(31.53%)	69(34.84%)
	Presence of semen	23 (33.82%)	38 (29.23%)	61(30.80%)
Comprehensive care practices for survivors	Testing the survivor	45 (66.17%)	96 (73.84%)	141(71.21%)
	Examining the survivor	48 (27.0%)	89 (68.46%)	137(69.19%)
	Treating the survivor	45 (66.17%)	106 (81.53%)	151(76.26%)
	Recording evidence of injuries	49 (72.05%)	60 (46.15%)	109(55.05%)
	Ensuring survivor safety	42 (61.76%)	81 (63.07%)	123(62.12%)
Challenges in providing care	Reporting and legal issues	57 (84.3%)	103 (83.07%)	165 (83.33%)
	Lack of specialized training for service providers	35 (52.0%)	94 (72.30%)	129 (65.15%)
	Geographical barriers (transportation)	23 (33.3%)	38 (29.23%)	61 (30.80%)
Triage priority for statutory rape Survivors	Disagree	55 (69.11%)	81 (69.23%)	136 (69.19%)
	Agree	12 (28.0%)	49 (41.1%)	61 (30.80%)
Primary reporters of statutory rape cases	Family members	57 (83.3%)	82(63.07%)	139 (70.20%)
	Religious leaders	6 (8.82%)	18(13.84%)	24 (12.10%)
	Police	21 (31.0%)	42 (32.30%)	63 (31.81%)

The reporting of statutory rape cases was predominantly carried out by family members (70.2%), followed by the police (32%) and religious leaders (12.1%). Doctors demonstrated significantly higher mean scores across all domains compared to nurses (Table 4). The mean knowledge score was 0.4449 ± 0.1508 for doctors and 0.3755 ± 0.1554 for nurses, with a significant mean difference of 0.0694 ± 0.0230 (t = 3.0130, p = 0.0029). Similarly, the mean attitude score was greater among doctors (0.5987 ± 0.1932) than nurses (0.5198 ± 0.1779), with a significant difference of 0.0790 ± 0.0274 (t = 2.8790, p = 0.0044). In terms of practice, doctors had a higher mean score (0.5593 ± 0.1761) compared to nurses (0.4779 ± 0.1818), with a significant mean difference of 0.0814 ± 0.0269 (t = 3.0225, p = 0.0028;). The overall KAP score followed the same pattern, with doctors scoring 0.5344 ± 0.1387 and nurses scoring 0.4573 ± 0.1387, resulting in a significant mean difference of 0.0771 ± 0.0207 (t = 3.7200, p = 0.0003). These findings indicate a statistically significant advantage in knowledge, attitude, and practice among doctors compared to nurses.

**Table 4:** knowledge, attitude and practice scores between doctors and nurses on statutory rape management in Ibadan (2024)

Variables	Doctor (mean ± SD)	Nurse (mean ± SD)	Difference (mean ± SE)	t-value	P-value
Knowledge score	0.4449 ± 0.1508	0.3755 ± 0.1554	0.0694 ± 0.0230	3.0130	0.0029
Attitude score	0.5987 ± 0.1932	0.5198 ± 0.1779	0.0790 ± 0.0274	2.8790	0.0044
Practice Score	0.5593 ± 0.1761	0.4779 ± 0.1818	0.0814 ± 0.0269	3.0225	0.0028
knowledge, attitudes, and practices score	0.5344 ± 0.1387	0.4573 ± 0.1387	0.0771 ± 0.0207	3.7200	0.0003

## Discussion

This study assessed the sociodemographic characteristics, knowledge, perceptions, and experiences of healthcare workers in Ibadan, Nigeria, regarding statutory rape. It also explored disparities between doctors and nurses and identified systemic barriers to effective case management. The findings highlight critical gaps in knowledge, attitudes, and practices that could significantly impact statutory rape case management. The sociodemographic analysis revealed that female healthcare workers comprised 74.2% of the sample, with nurses (60%) outnumbering doctors (15%). This reflects global trends where nursing remains a female-dominated profession due to historical gender roles and caregiving expectations [21]. Similarly, Adeyemi *et al*. [22] reported that female healthcare workers are more likely to engage in patient-centered roles, especially in managing cases of intimate partner violence and sexual assault. A notable concern was the higher proportion of nurses with less than five years of experience (38%) compared to doctors (18%). This suggests workforce instability, consistent with findings by Ayalew *et al*. [23], who reported that 50.74% of nurses in Sub-Saharan Africa intend to leave their jobs due to poor working conditions and limited career growth. High turnover rates among nurses may contribute to inconsistencies in statutory rape case management, as newer and less experienced nurses might lack specialized forensic knowledge. Income disparities were also evident, with doctors more likely to earn above ₦300,000 (19%) compared to nurses (3%). Ozumba and Alabere [24] attributed this to differences in educational requirements, specialization, and clinical responsibilities. Pay dissatisfaction remains a significant issue in the Nigerian healthcare sector, with only 16.9% of healthcare workers expressing satisfaction with their salaries. This dissatisfaction has been linked to absenteeism, low motivation, and workforce attrition [24]. Given that nurses had more hands-on experience with statutory rape cases, their lower salaries and high turnover rates could negatively impact case consistency and quality.

Although awareness of statutory rape was relatively high (81%), 25% of respondents either provided incorrect answers regarding the legal age of consent or expressed uncertainty. This finding aligns with Fouché *et al*. [25], who reported that 80% of South African community-service doctors were aware of statutory and have received some forensic training, although only 11.4% had hands-on exposure to rape cases during medical school. Additionally, 77.1% had never received training in medico-legal documentation, mirroring the knowledge gaps identified in this study. Similarly, Okonji *et al*. [26] emphasized that insufficient forensic training among Nigerian healthcare workers contributes to underreporting and inadequate documentation, weakening legal proceedings and case outcomes. A study by Laima *et al*. [27] on the management of rape cases at a specialist hospital in Gombe, Nigeria, further highlighted systemic challenges. Their findings showed that 67% of rape survivors did not receive medical care, 71.1% lacked psychological support, 42% were not given post-exposure prophylaxis (PEP) for HIV/AIDS, and 54% were not given antibiotic prophylaxis against sexually transmitted infections (STIs). These statistics underscore the urgent need for standardized protocols and enhanced training for healthcare providers handling sexual violence cases. Despite 76.3% of respondents acknowledging statutory rape as a serious issue, only 33.3% had managed such cases, with nurses (28.0%) having more hands-on experience than doctors (16.4%). This finding aligns with Rajesh *et al*. [28], who examined child sexual abuse law awareness among medical professionals in Puducherry, India.

Their study revealed substantial gaps in knowledge regarding mandatory reporting laws, medico-legal procedures, and survivor rights. Only (28%) of participants knew that doctors could examine a child sexual abuse survivor without a police requisition, and only (23%) knew that reporting sexual abuse cases to the police is mandatory. These gaps in legal literacy can significantly impact statutory rape case management. Systemic barriers to effective case management were also identified. The most frequently cited challenges included legal and reporting constraints (83.3%), lack of specialized training (65.2%), and geographical limitations (31%). Inadequate forensic documentation has severe legal consequences. Fouché *et al*. [25] found that improper rape case documentation often resulted in the release of alleged perpetrators due to insufficient evidence. Similarly, Laima *et al*. [27] reported that no treatment was documented for 38.3% of rape victims, and none returned for follow-up care. These findings highlight the need for structured forensic training and survivor-centered healthcare policies. The KAP analysis showed significant differences between doctors and nurses, with doctors scoring higher in knowledge (0.4449 ± 0.1508 vs. 0.3755 ± 0.1554; p = 0.0029), attitude (0.5987 ± 0.1932 vs. 0.5198 ± 0.1779; p = 0.0044), and practice (0.5593 ± 0.1761 vs. 0.4779 ± 0.1818; p = 0.0028). These findings align with studies in Tanzania [29] and Nigeria [30], where physicians demonstrated better competence in managing sexual violence cases due to greater access to training, while inadequate forensic knowledge among healthcare workers led to suboptimal interventions. Similarly, Rajesh *et al*. [28] found that most Indian medical professionals lacked knowledge of rape laws, reflecting broader medico-legal gaps.

**Limitation:** this study is limited by its focus on hospitals in Ibadan, restricting generalizability to other regions. The reliance on self-reported data introduces potential recall and social desirability biases, which may lead to overestimated knowledge or underreported experiences. Additionally, due to the stigma and legal complexities surrounding statutory rape, some cases may have been underreported, affecting the accuracy of the findings. Despite these limitations, the study provides valuable insights into healthcare workers' preparedness and highlights areas for improvement in statutory rape case management.

## Conclusion

This study reveals significant gaps in knowledge, attitude, and practice of healthcare workers in managing statutory rape in Ibadan, Nigeria. While most of the workers were familiar with statutory rape, many had inadequate knowledge of legalities, such as the age of consent and statutory rape laws. The doctors possessed greater knowledge and attitude compared to the nurses, although the nurses were more experienced in case management. However, the majority of the health professionals were inexperienced in managing such cases, indicating a gap between theory and practice. Based on the findings, it is recommended that health providers receive additional forensic and legal training to increase their ability to manage cases of statutory rape. Health facilities should also have established protocols for reporting and managing such cases, and an attempt should be made to improve collaboration between healthcare and legal institutions.

### What is known about this topic

- Statutory rape has significant physical, psychological, and social impacts on survivors, with healthcare professionals playing a critical role in their care;
- Inadequate training, poor legal awareness, and the absence of standardized protocols limit healthcare workers' ability to manage cases effectively;
- Weak collaboration between healthcare and legal systems further undermines justice and survivor support in low- and middle-income countries.

### What this study adds

- Reveals that among health workers in Ibadan, doctors have significantly higher knowledge, attitude, and practice compared to nurses, although nurses managed more cases;
- Uncovers systemic barriers including insufficient forensic training, challenges in case reporting, and societal stigma affecting statutory rape management in Ibadan;
- Highlights the need for targeted forensic and legal training interventions among emergency healthcare providers in Nigeria.
